# P13 Cost effectiveness of point of care diagnostics for AMR: a systematic review

**DOI:** 10.1093/jacamr/dlad066.017

**Published:** 2023-06-14

**Authors:** Abraham Tolley, Akhil Bansal, Becca Murerwa

**Affiliations:** School of Clinical Medicine, University of Cambridge, UK; Faculty of Medicine and Health, University of Sydney, Australia; School of Medicine, University of Nairobi, Kenya

## Abstract

**Objectives:**

Antimicrobial resistance (AMR) is a major threat to global health and is driven partly by overprescription of antibiotics. Point-of-care tests (POCTs) can reduce antibiotic prescribing by distinguishing different types of infections and the sensitivities of pathogens to antimicrobials. Although evidence for their clinical efficacy is mixed, they may be highly cost-effective interventions when their benefits in reducing the burden of AMR are considered. We aimed to systematically evaluate the cost effectiveness of different POCTs in reducing AMR for the first time to inform national and global health policies.

**Methods:**

A systematic review was conducted in line with the PRISMA framework, and the protocol was registered on PROSPERO. MEDLINE, PubMed, EMBASE, Cochrane Library and Google Scholar were searched from January 2000 to November 2022 for studies that (i) were trial- or model-based economic evaluations, (ii) focused on POCTs aimed at reducing AMR or inappropriate antimicrobial prescription, (iii) included a cost-benefit metric and (iv) were original research written in English with full text available. Methodological quality was assessed using the Consensus of Health Economic Criteria.

**Results:**

157 studies were retrieved of which 20 were included after screening. These included both trial (*n* = 9) and modelling-based (*n* = 11) evaluations. Most focused on POCTs for respiratory infections (*n* = 9) with sexually transmitted infections and febrile illness other common conditions evaluated. A majority of studies utilized C-reactive protein POCTs (*n* = 11) with others utilizing pathogen-specific PCR tests or procalcitonin. Most evaluated the cost effectiveness of POCTs at reducing antimicrobial prescribing, with only 3 studies considering the societal cost of AMR. Most studies found that POCTs reduced antibiotic use, but with varying cost effectiveness: 16 studies showed cost efficacy by the threshold or other standards stipulated by the study, with POCTs for group B streptococcus and urinary tract infections notably not found to be cost effective. The reporting and methodological quality of the included studies was mixed: good (4), moderate (13) and low (3).

**Conclusions:**

There is limited research on the cost effectiveness of POCTs in reducing AMR, and most studies do not account for the wider societal costs of AMR. Current forecasts estimate that AMR will cause 10 million deaths and cost the world 100 trillion USD per year in 2050: consequently, it is vital that evaluations of POCTs include the significant benefits from reducing AMR additional to the short-term benefits of avoided antibiotic prescriptions. Given the rising burden of AMR, such interventions merit further evaluation and integration into healthcare policy.

